# Systematic Review of Digital Interventions for Adolescent and Young Adult Women's Body Image

**DOI:** 10.3389/fgwh.2022.832805

**Published:** 2022-03-17

**Authors:** Ciara Mahon, Veya Seekis

**Affiliations:** ^1^School of Psychology, University College Dublin, Dublin, Ireland; ^2^School of Applied Psychology, Griffith University, Gold Coast, QLD, Australia

**Keywords:** digital, body image, systematic review, universal intervention, adolescent girl, young women

## Abstract

**Background:**

A growing number of digital interventions have been developed to address a range of mental health issues including body image. Identifying effective interventions for body image is important given the prevalence of body image concerns and their associated negative outcomes among young women. This review aimed to critically evaluate current evidence on the use of digital interventions for adolescent and young adult women's body image.

**Methods:**

A literature search was performed in November 2021 across three databases, PsychINFO, Web of Science and Pubmed to identify studies published with keywords and synonyms of “body image” and “digital interventions” that have been conducted with girls/women aged 10–25 years. Studies were included if they assessed a universal body image intervention delivered through a digital platform (e.g., web-based, mobile phone) and if they reported body image outcomes using validated measures. Two authors independently screened studies extracted data and assessed the quality of studies.

**Results:**

Eight of 15 interventions were effective in improving at least one body image outcome from pre-post intervention; however, effect sizes were mostly small-medium, and few effects were maintained at follow-up. Studies were heterogeneous in terms of delivery format, duration, sample characteristics, quality, and outcomes.

**Discussion:**

Findings suggest that digitally delivered interventions can effectively improve some body image outcomes in young women. Characteristics of effective studies are discussed and suggestions for future work on intervention development in this field are provided.

**Systematic Review Registration:**

https://www.crd.york.ac.uk/prospero/display_record.php?ID=CRD42021281435, identifier CRD42021281435.

## Introduction

Digital technologies and internet devices have become an integral part of young people's lives (1, 2) and, as such, could provide opportunities to access information about mental health care. Access to digital mental health via mediums including electronic devices such as smartwatches/phones and virtual reality headsets, software such as mobile applications (apps), and internet-delivered services such as counseling, may provide numerous benefits. For example, digital technology can reach individuals living in remote areas (3) or those who would otherwise not seek help for fear of stigmatization (4) and can provide easily accessible service in a time efficient way. Access to digital mental health care may be particularly useful to girls and women confronted with body image concerns. Given that very few girls and women at risk of eating pathologies seek face-to-face care due to feelings of shame or fear of stigmatization (5–7), such technologies may be particularly useful in reducing those barriers.

Body image can be defined as the perception of how one's body looks and feels. It is a multifaceted construct shaped by emotions and physical sensations that can vary relative to mood, experience, and environment (8) and can be both negative and positive. Given the promotion of largely unattainable appearance standards in Western society, many girls and young women experience concerns about their appearance (9). Body image concerns, or negative body image, may be assessed via primary outcomes such as body dissatisfaction, which is the subjective negative evaluation of one's shape, weight, overall appearance, or specific body parts (10) and drive for thinness, which entails perceptions, behaviors, and attitudes that pressure one to conform to the cultural ideal of thinness (11). However, assessment of body image concerns also comprises of secondary outcomes which may perpetuate links between predictors of negative body image, such as media messages, and primary outcomes. Secondary risk factors may include habitual monitoring of one's appearance from an observer's perspective (body surveillance), experiencing indignity about one's appearance (body shame) and social appearance anxiety, which is the fear of being negatively evaluated based on one's appearance (12–14). Secondary risk factors may also involve the internalization of societal appearance standards and comparison processes with these standards via media images and peers (15).

Body image concerns are risk factors for, and can be symptomatic of, eating disorders (11–13, 15). Notably, the peak onset of eating disorders for women is during adolescence and emerging adulthood (16–20 years), a phenomenon that has been attributed to stressful life events (16). During the COVID-19 pandemic, overall incidence of eating disorders increased by 15.3% in 2020, compared with previous years; an increase that occurred solely in girls and young women (17). In high income countries, anorexia and bulimia nervosa are a leading cause of disability-adjusted life years (DALYS) in adolescent girls aged 15–19 years (18) contributing to potential costs for individuals, families, and society. Moreover, risk factors such as internalization of appearance ideals, body dissatisfaction and dietary restraint have been reported to start in girls as young as five (19) and continue into young adulthood (20) suggesting that universal preventative interventions may need to start early.

Positive body image, however, is not simply low levels of negative body image, but rather the acceptance of favorable views of one's body. Primary positive body image outcomes include body appreciation, body image flexibility and functionality (21, 22). The construct is flexible, holistic, and protective, and moves beyond simple appearance evaluation and satisfaction to include respecting, honoring, loving, and accepting the body, including its unique characteristics that may differ from societal appearance ideals (22, 23). Positive body image is associated with higher life satisfaction and lower body dissatisfaction (21), and may offer protective effects against internalization of the thin ideal (24). Indeed, early adolescents (10–13 years), with positive body image, report that their appearance is characterized by a functional and accepting view of their bodies (25). Recent prevention studies have also shown that body appreciation and body image flexibility/functionality can be increased for up to 6 months in women (26–28), suggesting that digital preventative interventions should consider the inclusion of positive body image constructs.

According to Gordon (29) preventive measures fall under the categories of universal and selective. Universal eating disorder prevention includes all non-symptomatic individuals and addresses all levels of risk with the primary aim of reducing risk factors (e.g., body dissatisfaction) and strengthening protective factors (e.g., body appreciation). Selective eating disorder prevention addresses subpopulations who are pre-screened for eating disorder risk factors such as body dissatisfaction [e.g., (28)]. To date, many eating disorder prevention studies have been conducted to evaluate and improve selective prevention programs, while research on universal programs is limited (30, 31). One recent review by Schwartz et al. (32) revealed that there is evidence for the efficacy of universal prevention programs in reducing eating disorder risk factors in the short-term. Notably, out of 21 studies from that review, only one study included a measure of empowerment to assess positive body image, and only two of the studies assessed universal prevention with emerging adult women. Similarly, a review and meta-analysis by Chua et al. (33), found that universal eating disorder prevention interventions in children reduced internalization of appearance ideals and increased body esteem at post-test, but no studies assessed components of positive body image. Furthermore, Kusina et al.'s (34) systematic review found mixed support for the effectiveness of classroom-based interventions in reducing body dissatisfaction and an absence of studies seeking to promote positive body image among adolescents. Consequently, the universal prevention programs reviewed to date may not yet be optimized to include digital delivery and positive body image outcomes, whilst further investigation of these programs is required in young adult women.

Numerous systematic reviews and meta-analyses have been conducted on the use of digital technology to improve wellbeing (35, 36), and to treat/reduce eating disorder symptoms in “at risk” adolescents and young people [e.g., (37–39)]. However, a limited number of reviews have investigated digitally delivered universal interventions to mitigate body image concerns and enhance positive body image. Additionally, Bauer et al. (40) outlined a series of internet-based interventions aimed at preventing eating disorders, in young women, and concluded that based on the limited studies available, although feasibility and acceptability were promising, evidence on efficacy and effectiveness was limited. Similarly, Loucas et al. (41) revealed that despite inclusion of control groups and randomization procedures of the twenty preventative e-therapy studies reviewed, in samples of mostly women, conclusions could not be drawn on intervention effectiveness possibly due to the modest sample sizes.

Given the high prevalence of body dissatisfaction experienced by many adolescent girls and young adult women (42), and high levels of digital and internet device usage in this population (1, 2), the current narrative systematic review provides a synthesis of the current evidence on the effectiveness of universal prevention interventions delivered solely in digital format. In accordance with the age range for young people set by the World Health Organization (43) and body image researchers examining young adult women [e.g., (21, 26, 27)], the current review focuses on studies whose samples comprise 10–25-year-old females. Furthermore, given the growth of technology-based interventions and the publication of new studies since the last review on digital interventions in eating disorder prevention (41), this article focuses specifically on digitally delivered universal prevention studies aimed at improving primary (e.g., body dissatisfaction, body esteem, drive for thinness, body appreciation) and/or secondary (e.g., internalization of the thin ideal, appearance comparisons) body image outcomes in girls and young women.

## Methods

### Search Strategy

The review was conducted using a predefined protocol consistent with the guidelines for Preferred Reporting Items for Systematic Reviews and Meta-Analysis [PRISMA; (44)]. This protocol was preregistered on the Prospero database https://www.crd.york.ac.uk/prospero/display_record.php?ID=CRD42021281435. Searches on databases PubMed, PsycINFO, Web of Science were conducted between October and November 2021. Searches were limited to peer reviewed journal articles that were published in English. Non-peer-reviewed studies (e.g., dissertations, protocol, or process evaluation papers), were excluded as search and selection procedures for these studies can introduce bias (45). Book chapters, reviews, and qualitative papers were also excluded. There were no restrictions on publication date. A combination of key words that captured the target population age and gender characteristics, body image outcomes, intervention format and type, were used (see Supplementary Table 1). Identified articles were screened independently by two researchers (CM and VS) using Rayyan software.

### Eligibility Criteria

Eligibility criteria were determined by following a predefined PICOS (Population, Intervention, Comparator, Outcome, Setting) framework:

#### Population

Adolescent and young adult women aged 10–25 years were included. According to the World Health Organization (43) adolescents are defined as individuals aged 10–19 years, young people are aged 10–24 years. Yet, a vast majority of body image studies examining body image in young adults include participants aged up to 25 years (28, 46–48), hence studies with participants in this age range were included.

#### Intervention

This study included body image specific, mental health interventions, that were partially or fully self-administered/delivered through a digital platform (e.g., web-based, computer, or mobile phone). This study included universal primary prevention programs only; secondary selective programs targeted at individuals “at risk” of body dissatisfaction, treatment-based programs or programs for clinical groups were excluded. Interventions that were primarily face-to-face with the inclusion of some online technology as an element of the program were not included. Interventions that primarily focus on scars/burns/disfigurement, and/or young people with chronic disease or identified neurological or psychotic disorders, were not included.

#### Comparator

Active (i.e., standard non-digital care and alternative materials) or passive control (i.e., waitlist control and/or no treatment) were included as comparators. Studies that did not contain a control group (active or passive) were excluded.

#### Outcome

Changes in body image outcomes from baseline to last available follow up were the main outcomes for the review. Studies were included if they measured at least one body image variable as a primary outcome of interest. The concept of body image has been defined in various ways in the literature including body dissatisfaction, body esteem, body appreciation, body image flexibility, and body functionality, therefore each of these constructs were included in the review. Studies were excluded if they did not measure body image outcome variables or if body image outcomes were only included as a secondary outcome of interest.

#### Setting

Non-clinical, non-facility-based settings in any country were included. This review included studies that use a randomized control trial or quasi-experimental design. Pilot trials, which we define as studies that (a) do not compare to a control group, and (b) are primarily used to assess feasibility and acceptability of an intervention, were not included. Studies were excluded if they did not provide sufficient data to report significance outcomes and/or effect sizes for female participants.

### Data Extraction

Study information was independently extracted by two researchers, who each extracted one half of the data and checked the other half to verify that it was extracted correctly. A matrix with extracted data was developed and included: study characteristics (author, year, country), participant characteristics (age, sex/gender, ethnicity, sample size), intervention characteristics (study design, intervention and control types, delivery format, delivery medium, duration, measures taken) and intervention effectiveness (results, conclusions/outcomes, and implementation findings). Any discrepancies in data extraction were resolved via consensus by the two reviewers, with a third reviewer involved when necessary.

### Quality Assessments

Criteria from the Cochrane Collaboration's tool for assessing risk of bias (49) were employed to assess the quality of the studies. These criteria include: sequence generation, allocation concealment, blinding procedures, incomplete outcome data, selective outcome reporting and other sources of bias. Risk-of-bias assessments were performed independently by two researchers. For each of the domains, a rating of high, medium, medium to low, or low risk was assigned; where disagreements arose, discussions took place to resolve issues and reach consensus.

### Data Synthesis

Given the nascence of digital technologies for body image, and the heterogeneity of digital interventions for mental health in terms of content and delivery, a narrative synthesis, guided by the SWiM reporting guidelines (50) was used. We synthesized evidence from the articles that described the effectiveness of digital interventions against body image outcomes (e.g., change in body dissatisfaction, body appreciation), intervention approach used (e.g., cognitive dissonance, media literacy) and mode of delivery/digital platform (e.g., computer-based, smartphone-based) used. We also reviewed factors associated with effectiveness, sustainability of outcomes, completion, and adherence. In line with Campbell et al. (50) we undertook informal methods to investigate heterogeneity in findings by ordering tables by subpopulations (e.g., age, ethnicity, risk status), intervention type, delivery style and format, intervention duration, intervention components, and contextual/setting factors. To synthesize data on intervention effects we reported on *p*-values and summary statistics of intervention effect estimates.

## Results

**Figure 1:**
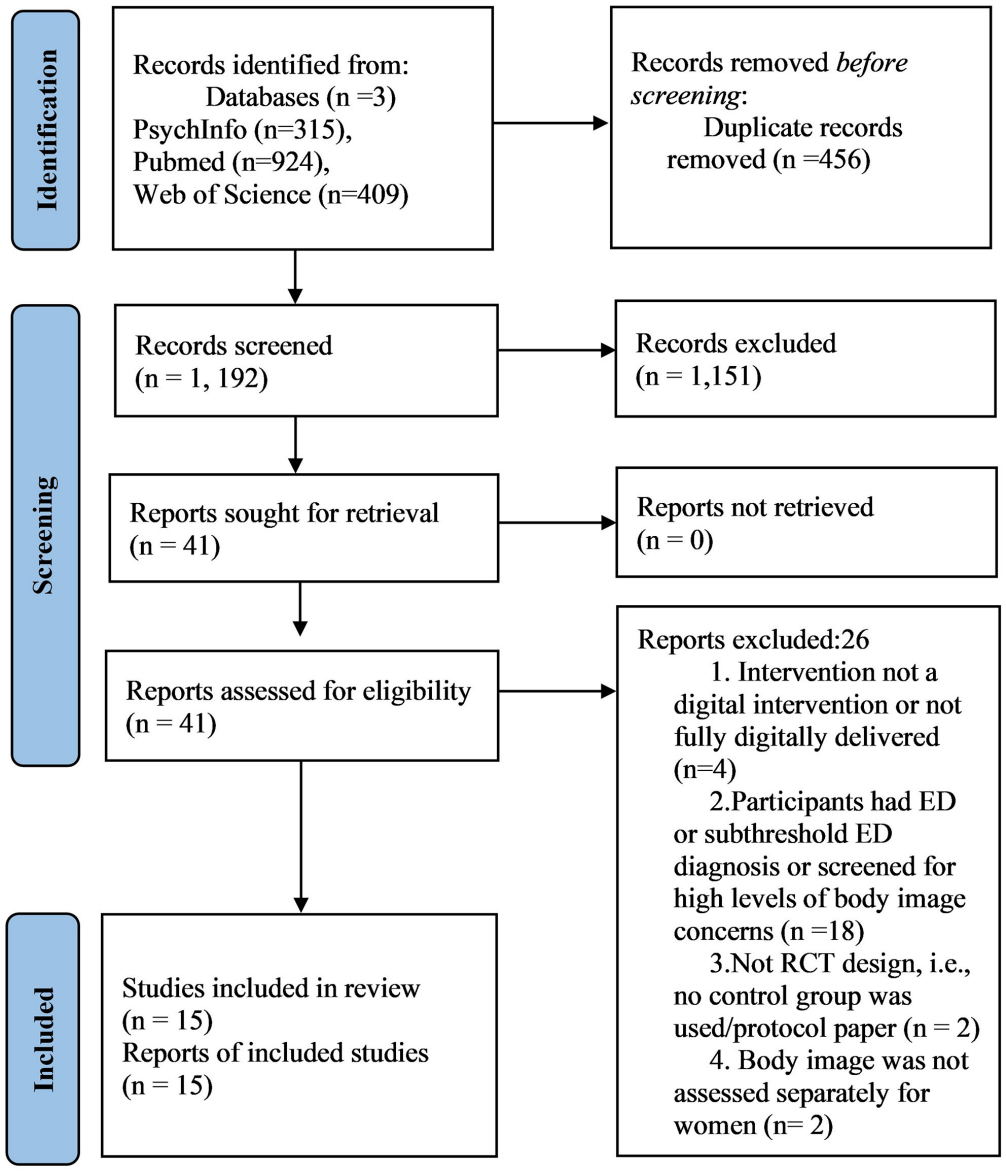
PRISMA 2020: flow diagram for systematic review.

The initial search yielded 1,648 results. After excluding duplicates there were 1,192 articles. Of these, 1,151 were excluded because they failed to meet inclusion criteria. Full text review was conducted on 41 articles, of which 26 were removed because inclusion criteria were not met (see Figure 1 for search strategy). A total of 15 studies were included in the final review (see Table 1 for characteristics of studies included in the review).

**Table 1:** Characteristics of studies included in systematic review.

**References “Name of intervention”**	**Country, N, M*age* (SD), ethnicity**	**Body image measure(s)**	**Intervention [study type, program type, delivery format/medium, duration]**	**Body image findings**
Alleva et al. (2018) (26) “Functionality based intervention”	• UK • 261 undergraduate women • M*age* = 22.79 (3.75) • 87.4% Caucasian	• Trait Body Satisfaction (BASS-MBSRQ) • State Body Satisfaction (VAS) • Body esteem (BES) • Body Appreciation (BAS-2) • Body Complexity (BCQ) • Body Self-Integration Scale (BSI)	• Experimental: • Functionality-based intervention (FBI) (*n* = 132) vs. active control (129) • Interactive (writing tasks) • Self-directed • Online. • Pre-test, 3 intervention lab-based sessions over week, post-test. Follow up: 1 week and 1 month.	Compared to control (creative writing), intervention group (Functionality writing) showed increases in trait appearance satisfaction, functionality satisfaction, and body appreciation at Post-test, 1 week and 1-month Follow-Up. The impact of media exposure on state body satisfaction did not differ for participants in the functionality vs. control groups.
Atkinson and Diedrichs (2021) (51) “Video-based micro-interventions”	• UK, • 202 undergraduate women, • M*age* = 20.79 (2.75). • 87.6% Caucasian.	• State Body image (8 VAS scales), • Body appreciation (BAS-2), • Body image flexibility (BIAAQ), • Appearance internalization & media pressures (SATAQ3-AI, SATAQ3-MP), • Shape & Weight concerns (EDEQ-WS, EDEQ-SS)	• Experimental • Mindfulness condition (MC) (*n* = 67) vs. Dissonance condition (DC) (*n* = 70) vs. Educational Control (*n* = 65). • Interactive (15 min videos & worksheet). • Video-based. • Researcher-led. • 1 lab session, Pre-post, 1-week follow up.	Both MC & DC reduced state internalization, perceived pressures and appearance distress vs. EC. DC reduced trait weight and shape concerns and internalization vs. EC. MC reduced appearance-ideal internalization only (small effect sizes). At 1 week follow up: MC and DC had improved trait body appreciation vs. EC (small effect sizes).
Bruning Brown et al. (2004) (52) “Student bodies”	• USA, • 152 high school girls, • Ma*ge* = 15.1 (.4), • 55.6% Caucasian.	• Weight concerns (EDEQ-WS) (WCS) • Shape concerns (EDEQ-SS) • Drive for thinness (EDI-DT)	• RCT: Dissonance-based Intervention (*n* = 102) vs. Wait List Control (WL) (*n* = 51). • Interactive. • Teacher-led. • Computer-delivered, in class. • 8 × 60 min sessions. 3-month follow up.	No significant differences on body image outcomes from pre-to-post or follow up in IG vs. WL groups.
Franko et al. (2012) (53) “Food, Mood, and Attitude (FMA) program”	• USA, • 64 undergraduate women, • M*age* = 20.18 (1.77), • 100% Latina	• Body dissatisfaction (BSQ), • Body ideal internalization (SATAQ3)	• Experimental • Psychoeducational Intervention (*n* = 32) vs. Control (browsing science-based websites) (*n* = 32). • Interactive. • Researcher and self-directed. computer-delivered • 2 × 2 h lab sessions over 4 weeks.	Body dissatisfaction marginally significantly reduced for intervention vs. control from pre-post.
Franko et al. (2013) (54) “Bodimojo”	• USA • 113 secondary school girls • M*age* =15.2,.78 • 33.5% African-American • 32% Caucasian	• Body esteem (BESAA) • Body dissatisfaction (EDI-BD) • Physical appearance comparison (PACS)	• RCT: Social cognitive/behavior change intervention & WL control (*N* of groups was not reported) • Interactive • Teacher led • Internet/website • Pre-test, 4–6-week post-test, 3-month follow-up	ITT: Relative to WL control, IG (Bodimojo) decreased in body dissatisfaction and appearance comparisons and increased body esteem at post-test (small ES) but not at 3-month follow-up
Fuller- Tyszkiewicz et al. (2019) (55) “Mindfulness & Gratitude Micro-interventions”	• Australia, • 247 women • Age = 18+ (no M/SD) • N/A Ethnicity	• Trait Body satisfaction (BISS) • State body satisfaction (VAS) • Body change (BICI) • Body image importance (BIIS)	• Experimental • Mindfulness & gratitude intervention (*n* = 147) vs. WL control (*n* = 100) • Interactive (video & exercises) • Self-directed • 11 (2–3-min videos) • Pre-post (21 days)	Compared to WL control, body satisfaction significantly improved by post-intervention for the intervention group (*d* = 0.42, moderate effect size) However high attrition rates.
Halliwell et al. (2011) (56) “Dove's ‘Evolution video'”	• UK, • 127 secondary school girls, • M*age* = 11.6 (1.1), • N/A Ethnicity	• Trait body dissatisfaction (EDI-BD) • State body satisfaction (BISS) • State body esteem (BES)	• Experimental • Psychoeducation Intervention (PI) & thin ideal exposure (*n* = 37) vs. PI & Control image exposure (*n* = 30) vs. No PI & Thin ideal (*n* = 31), No PI & Control image (*n* = 29).	In the intervention group (shown video), no significant differences in body image and body esteem reported when exposed to thin ideals vs. control images. In no intervention group, body image and body esteem were lower when exposed to thin vs.
			• Didactic. • Researcher. • 1 min psychoeducation video. Pre-post.	control images. Video may have protective effect for body image.
Kosinski (2019) (57) “Evaluative conditioning (EC) app”	• France, • 60 undergraduate women, M*age*=19.55 (1.36) • N/A Ethnicity	• Body dissatisfaction (EDI-BD) • Drive for thinness (EDI-DT) • Body satisfaction (CDRS)	• Experimental: EC condition app (*n* = 30) vs. Neutral condition (*n* = 30), • Interactive (EC pair photographs of their own body with positive photographs, neutral condition pair photographs of their own body with neutral photographs), • Self-directed • App-based i-pad delivered, • 1 game p/d, 7 days. (pre-post:1 week).	Body dissatisfaction and drive for thinness reduced from pre-to-post across both conditions, but no difference between EC or Neutral conditions (small-med effects).
Low et al. (2006) (58) “Student Bodies”	• USA • 72 undergraduate women (1st & 2nd year) • Age breakdown = N/A • 91.6% Caucasian	• Trait Body dissatisfaction (EDI-BD) • State Body dissatisfaction (SFRS) • Drive for thinness (EDI-DT) • Body Ideal Internalization and body image awareness (SATAQ) • Weight concerns (WCS)	• RCT • Student Bodies (SB), with a clinically moderated discussion group vs. SB unmoderated discussion group vs. SB no discussion group vs. control • Self-directed • Interactive • Online • 8-week intervention, 1 month follow up	No significant reductions in drive for thinness or body dissatisfaction or weight concerns between the intervention and control group at post-test. At follow up, unmoderated groups showed lower body dissatisfaction than controls (*p* = 0.03). Time spent using SB was significantly correlated with decreasing drive for thinness [*r* = 0.25 (57), *p* < 0.05] and lower reported weight and shape concerns at long-term follow-up.
Matheson et al. (2020) (59) “Micro-intervention animations”	• USA • 666 girls 7–14 years • M*age*=10.53 (1.11) • 54% White	• State body satisfaction state (VAS) • Trait body satisfaction (CFRS)	• RCT • Appearance teasing & bullying animation (*n* = 119) vs. Media & Celebrities animation (*n* = 113) vs. Control animation (*n* = 99). • Didactic. • Research-led. • Video (micro-intervention) • Pre-test, viewed 60 s animation (in lab) then 1 week later assessed post-test (at home online)	No differences in state or trait measures of body image in girls from pre-post intervention across conditions. However, girls 7–10 years, but not 11–14, in all animation conditions experienced significant moderate improvements in state body satisfaction.
Matheson et al. (2021) (60) “Playable technologies”	• UK • 3,354 girls aged 13–14 years • M*age* not reported • 81.6% Caucasian	• State body satisfaction (VAS) • Body esteem (BESAA). • Internalization of media ideals (SATAQ-3)	• RCT • Psychoeducational & Cognitive Dissonance Body image playable (*n* = 1,093) vs. Psychoeducational Body Image Social media posts (SNS) (*n* = 895) vs. Environment Conservation playable (Control) (*n* = 1, 197) • Researcher • Playable psychoeducational game • Pre-post (immediately after viewing playable in lab)	For girls in the body image playable and SNS conditions significant improvements were shown in body satisfaction at post-intervention relative to the conservation playable. The body image playable did not protect against the negative effects associated with viewing idealized media images.
Mulgrew et al. (2019) (61) “Body Functionality Intervention”	• Australia • 117 college students, • M*age* = 23.48 (1.36) • 91% Caucasian	• State satisfaction, functionality satisfaction (16 VAS) • Body appreciation (BAS-2) • Body esteem (BES) • Self-objectification (SOQ)	• Experimental: Functionality-based intervention (FBI) (*n* = 54) vs. Stress Management intervention (control) (*n* = 63) • Interactive (writing tasks) • Self-directed • Pre-test (2 sessions, 1 week), Post test	No significant differences between FBI and control body image outcomes. Within sessions changes: Session 1. appearance satisfaction increased in both conditions, but more so in FBI. Session 2 [receipt of FBI and control sessions], participants experienced significant
				improvements in state appearance satisfaction, functionality satisfaction, body appreciation. Women in the FBI program experienced a larger increase in body appreciation than women in the control group.
Serdar et al. (2014) (62) “Dissonance based program”	USA, 133 undergraduate women, M*age* = 18.83 (1.38), 43.5% Caucasian	Body ideal internalization (IBSS-R) Body esteem (BES)	RCT: Face to face (F2F) dissonance (n=107) vs Online dissonance (n=112) vs Control (n=114),. Interactive Delivered via website Self-directed & trained doctoral students 3x1hr text-based group discussions Pre-post, (4 weeks)	Improvements in body esteem in both F2F and online, but not control from pre-post. Improvements remained significant after controlling for dose in both active conditions. Active conditions did not differ from each other. Session attendance was significantly associated with outcomes for all active condition participants.
Toole and Craighead (2016) (63) “Self-compassion meditation training”	• USA, • 80 undergraduate women, • M*age* = 18.85 (0.87) • 50% Caucasian, • 30% Asian American.	• Body appreciation (BAS) • Body surveillance (OBCS) • Body shame (OBCS) • Body dissatisfaction (BSQ)	• Experimental: Self-compassion meditation podcast (*n* = 40), WL control (*n* = 40) • Interactive. • Self-directed. • Audio-delivered podcasts. • Pre-test (20-min podcast per day for 1 week) Post-test.	ITT: Compared to control, intervention group increased in body appreciation and decreased in body surveillance, appearance contingent-self-worth, at post-test (small ES). No difference in body shame and body dissatisfaction between groups at post-test. Meditation practice frequency was not associated with change in body image outcomes.
Winzelberg et al. (2000) (64) “Student Bodies”	• USA, • 60 undergraduate women, • M*age* = 20 (2.8), • 53% Caucasian	• Body dissatisfaction (BSQ), • Drive for thinness (EDI-DT), • Weight & Shape concerns (EDEQ-WS; EDEQ-SS).	• RCT • Dissonance-based intervention (*n* = 31) vs. Control (*n* = 29). • Interactive. • Self-directed. • Computer-delivered • 8 × 90 min sessions. 3-month follow up.	ITT analyses: No differences in body image outcomes between intervention group (IG) and control at post-test. At follow up, body image and drive for thinness improved in IG vs. controls.

*BAS, Body Appreciation Scale (65); BAS-2, Body Appreciation Scale-2 (66); BSQ, Body Shape Questionnaire (67); BSQ-SF, Body Shape Questionnaire-Short Form (68); BIAAQ, Body-Image Acceptance and Action Questionnaire (69); OBCS, Objectified Body Consciousness Scale (70); BESAA, Body Esteem Scale for adolescents and adults (71); BES, Body Esteem Scale (72); BISS, Body Image Satisfaction Scale (73); BICI, Body image change inventory (74); BIIS, Body Image Importance Scale (75); BSI, Body-self-integration scale (76); BASS-MBSRQ, Body Appearance Satisfaction Scale of the Multidimensional Body-Self Relations Questionnaire (77); BCQ, Body Complexity Questionnaire (78); CFRS, Child Figure Rating Scale (79); EDEQ, Eating Disorder Examination Questionnaire (EDEQ-WS, Weight concerns subscale; EDEQ-SS, Shape concerns subscale of the EDEQ) (80); EDI-DT, Drive for thinness subscale of Eating Disorder Inventory; EDI-BD, Body dissatisfaction subscale of Eating Disorder Inventory (11); WCS, Weight Concerns scale (81); PACS, Physical Appearance Comparison Scale (82); SATAQ-3, Sociocultural Attitudes Toward Appearance Questionnaire 3 (83) [AI, appearance internalization subscale; MP, media pressures subscale]; SATAQ-4, Sociocultural Attitudes Toward Appearance Questionnaire 4 (84); VAS, Visual Analog Scales (85); SOQ, Self-objectification Questionnaire (86); SFRS, Stunkard Figural Rating Scale (87); BDS, Body dissatisfaction scale (88); ITT, intention to treat analysis*.

Of the 15 studies included in the review eight were effective (i.e., they produced statistically significant changes in body image outcomes in experimental vs. control groups from pre to post intervention (26, 51, 53–55, 60, 62, 63). Of these, five reduced negative body image (53–55, 60, 62), two improved positive body image (51, 63) and one succeeded in both reducing body dissatisfaction and improving body appreciation (26).

Although these eight provided evidence of the effectiveness of the intervention, one study suggested the intervention had protective effects against media exposure within experimental groups only, however no pre-post changes in body image outcomes were reported between groups (56). Other studies improved body image from pre-to-post intervention, but failed to yield protective effects against media exposure, suggesting that effectiveness may have been somewhat limited [e.g., (60)]. Additionally, the changes reported by Franko et al. (53) were only marginally significant and the significant effects observed by Fuller-Tyszkiewicz et al. (55) may have been biased by the considerable attrition rate. Similarly, the significant findings observed by Serdar et al. (62) may have been colored by selective reporting bias, where only significant effects were reported. Furthermore, effect sizes across studies tended to be small-medium.

Few studies captured follow up data (*n* = 6); those that did, follow up durations were short (ranging from 1 week to 3 months). Few maintained effects at long term follow up, particularly at longer 3-month follow ups (52, 54). Improvements in body image outcomes at follow-up were reported by two studies that found no significant differences from pre-post (50, 64); while this could indicate delayed intervention effects, these findings should be interpreted cautiously. Atkinson and Diedrichs (51) reported maintenance effects for body image outcomes at 1 week follow up, while Alleva et al. (26) reported maintenance effects at 1 week and 1 month follow up. These findings suggest that some digitally delivered body image interventions may be effective, at least in the short term at improving body image; however, given the heterogeneity in these findings, there is a need to parse out elements of these interventions to ascertain which aspects may be particularly effective in improving women's body image.

### Characteristics of Program

#### Program Type

Dissonance, psychoeducation, functionality, mindfulness and self-compassion were among the more common intervention types employed. Cognitive dissonance, which involves critiquing body ideals to reduce pursuit of these ideals and body dissatisfaction (89), was investigated in six studies. Three of these were the “Student bodies” dissonance program, which were not found to reduce body dissatisfaction or drive for thinness (52, 58, 64). However, other dissonance approaches were found to improve body esteem (62), while micro-interventions grounded in cognitive dissonance improved body appreciation (51) and body satisfaction (60) although effect sizes were typically small.

Psychoeducational approaches, which involve educating individuals about aspects of body image, were employed in five studies, but varied in terms of content and were often delivered in conjunction with other approaches. There was mixed support for psychoeducational approaches with marginal-small reductions in body dissatisfaction reported by some studies at post-test but not at follow up (53, 54), and no effects observed in others (59). Matheson et al. (60) and Halliwell et al. (56), who delivered psychoeducation via short “playable” games, social media posts and educational videos also found that psychoeducation had some potential to improve body image particularly in younger adolescents/children.

There was mixed evidence for functionality-based interventions (*n* = 2) which encourage individuals to focus on the functionality of their bodies (what their bodies can do, vs. how they look). Mulgrew et al. (61) observed no overall improvements in body image outcomes in experimental vs. control groups, while Alleva et al. (26) found that functionality writing exhibited improved appearance satisfaction, functionality satisfaction and body appreciation with effects (small) maintained at follow up.

Two studies investigated mindfulness interventions [*n* = 2; (51, 55)], which encourage a non-judgmental awareness and acceptance in the present to disrupt sociocultural influences on body image and promote healthy body image. These were found to be effective in reducing body dissatisfaction and increasing body appreciation (small effect sizes). One study evaluated a self-compassion intervention, which involves directing warmth and kindness to disrupt self-critical thoughts associated with body dissatisfaction (63). This was found to be successful in improving body appreciation, but not reducing body dissatisfaction.

#### Delivery Format

Most interventions were interactive and included written, listening, or reading activities/worksheets, group discussions, online discussion boards and homework/assignments. There was mixed evidence for structured internet-based approaches containing lesson content, activities, personalized feedback, engagement reminders and discussion forums; while Franko et al.'s, (53, 54) psychoeducation interventions, and Serdar et al.'s (62) dissonance intervention, were found to improve body image, some [e.g., (52, 58, 64)] were not. Interventions that singularly incorporated thought exercises, writing and/or mindfulness/breathing in response to video content, were mostly effective in improving body image [e.g., (51, 55, 63)]; however similar, didactic approaches (*n* = 2) were more limited in their capacity to improve body image (56, 59). This suggests that interactive approaches may be more appropriate for improving body image compared with didactic approaches, but given mixed results, further research is required identify which delivery format is more appropriate for improving body image.

#### Delivery Medium

A variety of delivery mediums were used including computer/internet-based interventions (*n* = 9), audio or video clips (*n* = 4), and app-based games (*n* = 2). Many of these interventions reflect traditional body image intervention delivery formats (e.g., functional writing exercises/discussion groups), just delivered via a digital format (typing exercises/online discussion boards). While many computer/internet-based and audio/video-based interventions were effective, it is unclear whether these digital approaches are more/equally/less effective than traditional face to face approaches; one study (62) compared online vs. face-to-face delivery of a dissonance-based intervention, and found that both intervention groups improved body image, but no differences in outcomes between online and in person groups were observed. This could suggest that online delivery may be as effective as face-to-face delivery, at least for group sessions moderated in person or via the internet, but further research is required.

Several innovative delivery mediums were observed in this review. Of note, one study, Kosinski (57) investigated the effectiveness of a mobile app-based intervention that made use of gamification principles to improve body image; participants in the experimental “evaluative conditioning” condition, were required to pair photographs of their own body with positive photographs, and those in the neutral condition were required to pair photographs of their own body with neutral photographs. Participants received feedback on performance, points were awarded for correct performance and difficulty increased as participants proceeded through the task. Although no differences were observed between experimental and control groups, body image improved in both conditions from pre-to-post suggesting that gamified apps show some potential for improving body image.

Matheson et al. (60) explored the effectiveness of body image “digital playables”, which are in-app advertisements that make use of interactive mini-games, to impart body-related psychoeducation and media literacy to users. Players navigated a cartoon character through an online environment by swiping and tapping on the screen, while psychoeducational messages appeared. These digital playables significantly improved participant's body satisfaction, however, a social media control condition (which contained the same psychoeducational/media literacy messages as the digital playable but delivered in a static rather than interactive context) was found to be equally effective in improving body satisfaction. No studies included in the review explicitly investigated social media as a digital intervention medium, however, given this finding, social media could represent another potential useful delivery medium. The use of podcasting was another unique medium that was effective in improving body appreciation (63). Overall, digital formats appear to be an effective medium through which body image interventions can be delivered.

#### Delivery Setting and Program Interventionist

Studies were conducted in lab settings (*n* = 4), classroom settings (*n* = 2) or self-directed “at home” (*n* = 9). In most studies, participants self-administered interventions, however some interventions were delivered/led by researchers or aspects of the program (e.g., group discussions) facilitated by trained graduate students/researchers. Tightly controlled, lab-based and researcher-led studies tended to report significant results, while findings for classroom-based and self-directed “at home” settings were more variable. Nonetheless, findings do provide support for the use of self-directed digital interventions to improve body image outcomes, as four out of the nine effective interventions, were self-directed (53, 55, 62, 63). This could represent low-cost, accessible, and scalable means of delivering universal body image programs.

#### Comparator

Most interventions were evaluated against waitlist or passive control groups (*n* = 9), and of the studies that used passive controls, five were found to be effective (53–55, 62, 63). Although fewer studies used active controls (*n* = 5), there was some evidence that interventions yielded significant improvements in body image outcomes when active controls were used. For example, one study compared mindfulness and dissonance micro-interventions vs. an active educational control and found that both approaches significantly improved body appreciation, but the dissonance condition yielded change in more body image outcomes vs. the mindfulness condition (51). Another study which compared the effects of exposure to SNS post and a body image playable, vs. an active control (a conservation playable) and found that both the SNS post and body image playable yielded significant improvements in body satisfaction vs. the neutral active control (60). However, given that intervention effects often seem larger when compared to a passive/waitlist control group compared to an active control, future studies should seek to incorporate more active controls to avoid overestimating intervention effects.

#### Duration

Many studies involved multiple sessions that took place over several weeks (*n* = 6); however, longer duration sessions were not necessarily more effective than shorter duration sessions. In fact, some of the longer 8-week (52, 58, 64) and 4-week interventions (53) were not found to be very effective in improving body image. Most interventions (*n* = 9) were short duration, micro-interventions, where participants were exposed to materials briefly (between 1 and 20 min) for a single session or multiple short sessions over a week/2-week period. Relative to a control, some single session interventions (51, 60), and repeated short sessions over a limited time period (26, 63), were found to be effective, while others were not (57, 59, 61). Findings suggest that short-duration interventions may have the capacity to improve body image, at least over the short term.

#### Adherence/Engagement

While information regarding engagement/adherence/compliance to the intervention were not reported by some studies, studies that did report this, typically observed higher levels of compliance at the beginning of intervention and lower levels of compliance toward the end (58, 64). Adherence/engagements appeared to influence outcomes in some studies, but not others; some studies found that session attendance or time spent engaging with active conditions was significantly associated with participant body image outcomes [e.g., (58, 62)], while others reported no association between engagement levels and outcomes [e.g., (63, 64)]. However, there was a lot of variation in the degree of self-directed sessions, homework activities, and group discussion required of participants, therefore, it is unclear whether or to what extent, adherence/engagement levels influenced outcomes.

### Characteristics of Participants

#### Age

Most studies were conducted with undergraduate women, with only five studies conducted with adolescents (52, 54, 56, 59, 60). Interventions conducted with adolescents, included girls aged 15 years or younger, with no programs for those aged 16–18 years. Some programs targeting college-aged women were effective (26, 51, 53, 55, 62, 63) while others were not. Similarly, there was mixed evidence regarding the effectiveness of programs that targeted younger girls/adolescents.

#### Ethnicity

Although many countries were represented in this review, most studies emerged from the USA, UK, and Australia. While ethnicity was not reported in some studies (*n* = 3), most studies were conducted predominantly with Caucasian participants, except for one study that included Latina participants only (53). However, several studies reported diverse samples [e.g., (52, 54, 59, 62–64)]. Studies conducted with racially homogenous and heterogenous groups yielded similarly mixed results in terms of intervention effectiveness. However, one study found that ethnic minority groups experienced heightened benefits from a digital body image intervention (54). Findings suggest that digital body image interventions can be effective when conducted within both homogenous and heterogenous ethnic groups.

#### Risk Status

Although this review excluded interventions targeting “at risk” groups (see Supplementary Materials for full list of excluded studies), some studies assessed the impact of baseline characteristics on body image outcomes. Matheson et al. (60) found that girls with lower trait body esteem at baseline reported significantly greater improvements in body satisfaction, relative to those with higher esteem, while Franko et al. (54) found that girls with overweight BMI status reported greater reductions in body dissatisfaction from pre-post intervention. This suggests that individuals who may be more vulnerable to body image concerns, may experience greater benefits from body image programs.

### Quality Assessment

A Cochrane Quality assessment was conducted independently by two researchers on all included studies (see Supplementary Table 1, for full quality assessment table). Overall, the quality of studies was low (although there were some exceptions; see Table 2). While most studies appropriately randomized participants to conditions, concealment of this allocation was generally poor, which may have introduced selection bias. The risk of performance bias (due to knowledge of which intervention group they were assigned to) and detection bias (due to knowledge by researchers of which intervention group participants were assigned to) were mostly high. Risk of attrition bias was generally low, although some studies reported higher levels of attrition than others. Reporting bias also varied; some studies appeared to report all outcomes, however other studies failed to report effect sizes or only reported on significant findings. Other biases that were evident across studies included sampling bias; most studies involved convenience samples of college or school students, thereby limiting the generalizability of findings. Some studies handled missing data using multiple imputation techniques (26, 59, 60), while other studies failed to report on how missing data were handled. Furthermore, no study included in the review was preregistered and only two conducted power analyses to determine whether their sample size was sufficient to detect significant effects (26, 51), therefore it is possible that other studies may have been underpowered to detect effects. Given the varied quality of the studies included in the review, findings must be interpreted cautiously.

**Table 2:** Cochrane quality assessment table.

	**Selection bias - Random sequence generation**	**Selection bias - Allocation concealment**	**Performance bias - Blinding of participants**	**Detection bias - Blinding of outcome assessment**	**Attrition bias - Incomplete outcome data**	**Reporting bias**
Atkinson and Diedrichs (51)	M	H	L	H	L	L
Winzelberg et al. (64)	H	H	H	H	L	L
Bruning Brown et al. (52)	H	H	M	H	H	H
Halliwell et al. (56)	L	L	M	H	L	L-M
Serdar et al. (62)	L	H	H	H	L-M	H
Kosinski (57)	L	L	M	H	L	L
Low et al. (58)	L	H	H	H	L	H
Franko et al. (53)	L	M-H	H	H	L	L
Mulgrew et al. (61)	L	H	H	H	L-M	M
Toole and Craighead (63)	L	H	M	H	L	L
Franko et al. (54)	L	H	H	H	M-L	L
Matheson et al. (59)	L	L	H	H	L	L
Alleva et al. (26)	L	L	M	H	H	L
Fuller-Tyszkiewicz et al. (55)	L	L	H	H	H	L
Matheson et al. (60)	L	L	L	H	M-L	L
L	low risk					
M	medium risk					
H	high risk					

### Interrater Reliability

Researchers independently screened initial searches and demonstrated a 91.4% agreement/concordance level on studies to be included/excluded from the review (see Supplementary Table 2). For the quality assessment, a selection of studies (*n* = 6, 40%) were independently assessed by researchers and there was a 90.6% agreement level on study quality.

## Discussion

This narrative systematic review aimed to evaluate the effectiveness of digitally delivered universal eating disorder prevention interventions in improving primary (e.g., body dissatisfaction, drive for thinness, body appreciation) and/or secondary (e.g., internalization of the thin ideal) body image outcomes in girls and young women. The small number of studies identified in the literature search (i.e., *n* = 15) highlight that research in this field is novel, however, findings indicate that digitally delivered programs show promise in decreasing eating disorder symptomatology (e.g., body dissatisfaction, drive for thinness) and increasing aspects of positive body image (e.g., body appreciation) in girls and young women.

Notably, five of the intervention studies reliably reduced negative body image (53–55, 60, 62), two improved positive body image (51, 63), and one improved both body dissatisfaction and positive body image (26), in the short term, with effect sizes ranging mostly from small to medium. Secondary body image outcomes including internalization of appearance ideals and appearance comparisons were assessed by fewer studies but improvements in appearance comparison were shown at post-test (54) and internalization of appearance ideals at 1-week follow-up (51) with small effect sizes.

Regarding the follow-ups, only four of the studies in this review investigated between 1- week and 3-month periods. One study found sustained improvement in body appreciation at 1-week follow-up with small effects sizes (51), while another maintained increases in trait appearance satisfaction, functionality satisfaction, and body appreciation at 1-week and 1-month follow-up with negligible effect sizes (26). However, other studies did not yield reliable maintenance effects at 3-month follow up (54, 64). Given the limited studies with follow-up measures we cannot draw any conclusions into the sustainability of digitally delivered universal eating disorder prevention programs. Future research should strive to address the paucity of follow-up measures in universal interventions to provide insight into sustainability of effects, potentially determine beneficial timing of booster sessions, and whether the magnitude of post-intervention effects remain stable.

The current review aligns with, and contributes to, findings from recent reviews of mostly face-to-face universal eating disorder prevention programs (32, 34, 39), in two important ways. First, in line with prior reviews of face-to-face interventions, digital delivery of universal interventions yielded small to moderate effect sizes indicating they may be effective in reducing eating disorder risk factors among young adult women and adolescent girls. Thus, digital platforms may be useful and cost-efficient modes of delivery to facilitate access to universal interventions for girls and women, whether they are in school or campus settings, remote areas, or wish to use the program in the privacy of their own environment. Although one study in the current review (62), demonstrated that face-to-face and digital delivery of a universal dissonance-based program yielded comparable increases in body esteem among young women relative to a control, further research comparing both delivery modes is required to establish this comparability. Second, the current review suggests that digital delivery of some universal programs may provide a constructive avenue for strengthening aspects of positive body image, such as body appreciation (26, 51, 63). Given the growing emphasis on promoting strengths-based aspects of mental health and body image, the finding that digital interventions can promote positive body image is encouraging (22).

Although promising, the limited number of studies, and heterogeneity of intervention approach, design and digital platform make the conclusions of the review tentative. Various theoretical approaches were employed to improve body image. In line with previous reviews (32, 34, 90), most studies in the current review, used cognitive-dissonance strategies, while others utilized gratitude, mindfulness, and/or media literacy approaches, all of which yielded improvements in some body image outcomes with mostly small to medium effect sizes. Findings suggest that various theoretical approaches may be administered via digital platforms and or devices with beneficial body-related outcomes.

A further differentiating factor between the studies was the depth and duration of content delivery. Studies in this review administered digital interventions using one of two methods: standard (e.g., instructional guide for use over a period) or micro-intervention. Micro-intervention approaches differ from standard intervention programs in that they are designed to administer quickly consumed resources which may have an immediate positive impact on targeted symptoms (55). Indeed, many of the recent studies included in this review employed micro-interventions [e.g., (51, 55, 60)]. Moreover, digital micro-interventions can use different modes of technology such as brief animated films (59) and playables (60), which are in-app interactive advertisements that take a few minutes to complete. Based on their quick consumption of content, micro-interventions may be best used in-the-moment particularly when girls or women experience state body-related fluctuations. For example, Fuller-Tyszkiewicz et al. (55) found that although the micro-intervention increased body satisfaction in women, the strongest effects were found when state body satisfaction levels were lower. Additionally, interactive micro-interventions that required active engagement with content [e.g., (60)], appeared to be more effective than didactic/passive micro-intervention styles [e.g., (59)]. Although the digitally delivered micro-interventions in this review show promise in improving some body image outcomes, attrition rates were mixed. Future research will need to assess duration of, and engagement with, micro-interventions, particularly those using short films and playables, to establish support for their use in universal programs. Similarly, standard methods using multi-session instructional delivery (e.g., 8- or 4-weeks) which have shown improvements in body image [e.g., (54, 63)] require further replication to assess reliability of efficacy in universal programs using digital platforms.

Relatedly, use of digital platform varied between the studies. Most studies used a video format whereby participants accessed a video during class [e.g., (56)] in a lab session [e.g., (51)], or via a website, software, or smartphone app [e.g., (54, 55, 60)]. Other participants were sent a link to complete online activities [e.g., (26, 53)] or instructed to listen to a meditation podcast (63). Only three studies provided detailed participant evaluations of the digital platforms/devices used. Franko et al. (54) reported that program features with physical activity tracking, quizzes, and games scored the highest satisfaction ratings while Fuller-Tyszkiewicz et al. (55) found that participants rated usefulness of the app as moderately positive, and although just over half the participants from the Toole and Craighead (63) study would recommend the podcast to others, only 39% indicated a willingness to continue listening to the podcast. Inclusion of evaluation assessment is strongly recommended when conducting digital interventions to better understand the useful (and challenging) aspects of accessing and interacting with content on the platform/device.

A noteworthy finding from one study was that viewing psychoeducational playables was equally effective at enhancing body satisfaction in adolescent girls as viewing psychoeducational content on social media posts. Furthermore, relative to playables, viewing posts on social media platforms perpetuated significantly greater prosocial body image behavior such as uploading unedited photos (60). As noted by Matheson et al. (60) prosocial behavior is under-researched in the body image literature. Given the ease of social media use for young people further research could investigate whether body image prosocial behaviors can be encouraged through social media platforms to improve body image in girls and young women.

A further issue regarding dissemination of interventions via digital platforms is the level of involvement of moderators, researchers/experimenters, teachers, and other healthcare professionals. Indeed, the amount of professional time and/or training required to deliver or moderate digital programs may impact sustainability and cost. Most of the studies in this review were researcher (or teacher) led or monitored, some were self-directed (or a combination of researcher led and self-directed) and two were interactive (i.e., included discussion groups). All modes revealed improvements in some body image constructs. Importantly, as per the studies included in this review [e.g., (59)], whenever young children are exposed to digital content, researcher or teacher led interventions would be recommended, as dissemination of universal prevention interventions has shown to be successful in classroom settings (32, 34, 91). With regards to emerging adult women, future research could quantitatively examine whether a difference exists between moderator-led and self-directed digital universal interventions, however inclusion of in-depth qualitative participant feedback could provide deeper insight into their experiences of each modality. Alternatively, if professional input is found to be more useful than self-directed interventions, future research is needed to determine the optimal level of professional input. Another digital feature that may alleviate the costs associated with professional input is that of conditional branching, whereby a participant moves to a feature based on their state responses. Indeed, micro-interventions, which are intended for immediate impact, may be an ideal format to examine conditional branching and just-in-time-adapted interventions (JITAI), given the potential strength of digital interventions is the ability to reach those whom it would most benefit (92).

It is important to note that all studies were conducted in Western countries. Although one study focused on Latina college women (53) and two studies contained racially/ethnically heterogenous samples, most samples were White and included only a small percentage of non-white participants, thus, the obtained effects may not necessarily apply to non-white girls and women in non-western nations. Given that recent research indicates high body dissatisfaction prevalence rates in countries such as China (93) and Brazil (94), and the increasing global use of digital devices (95) further investigation of digitally delivered universal interventions in these and other non-Western populations is warranted.

Furthermore, while digital interventions were found to improve body image outcomes among adolescents, comparatively fewer studies were conducted with adolescents. Studies that did include adolescent samples, focused on girls aged 15 years and below, with no interventions targeting late adolescents (16–18 years). Although there is some evidence that larger effect sizes are obtained in body image research conducted with younger adolescents (91), adolescence and emerging adulthood are vulnerable times for body image, and it is pertinent that future studies address this gap and consider older adolescents when designing digitally-based interventions.

A methodological strength of most studies was the rigorous approach to design in terms of subject randomization, intention-to-treat analyses, and use of active control groups. Additionally, attrition rates mostly ranged from low to medium, suggesting that digital delivery may provide sustained content engagement. However, some studies were methodologically limited by short term intervention, high rates of attrition, short follow up, performance bias and report bias and sampling issues. Most studies were not pre-registered and failed to include a priori power analyses to indicate whether studies were sufficiently powered to detect statistical effects. Furthermore, most studies were conducted with convenience samples of university and/or school students, which may limit the generalizability of findings. Future studies should seek to address these methodological limitations to ensure research findings are robust. Additionally future intervention studies should incorporate formal process evaluations to identify mechanisms, barriers/facilitators and contextual factors underpinning intervention effects to inform further development and refinement of digital body image interventions (96, 97).

## Limitations

Several limitations of this review should be noted. Given the novelty of digital universal prevention interventions in body image and that our review was focused on girls and young adult women (10–25 years), only a limited number of studies could be included. Based on the exclusion criteria, it is possible that other digital interventions not included in this review could decrease body image concerns and increase positive body image [e.g., (98, 99)]. Moreover, the included studies used different types of intervention approaches, modes of delivery, and platforms complicating the process of reaching reliable conclusions. The search was also limited to universal prevention interventions in body image published in English across three databases, which may pose potential publication biases. Finally, because of time constraints we did not follow up with authors to source original data when insufficient data was presented in published reports. Consistent with the preregistered protocol as outlined in the methods section, two studies in this review were considered to provide incomplete data and were excluded, however, these studies are listed in Supplementary Materials (100, 101).

## Conclusion

Overall, findings suggest that digitally delivered body image interventions can reduce body dissatisfaction and improve body appreciation in young adult women and girls. Given the relative nascence of this research, further studies are required to replicate and extend on the findings of this review. Further research, using more diverse samples, younger age groups, longer follow up durations and rigorous research designs are required. Research also needs to ascertain participant feedback on the elements of digital interventions that enhance adherence, engagement, acceptability and effectiveness, as well as the appropriate dose and duration, so as to maximize intervention effects.

## Data Availability Statement

The original contributions presented in the study are included in the article/Supplementary Material, further inquiries can be directed to the corresponding author/s.

## Author Contributions

CM and VS conceptualized the study, wrote the study protocol, screened and extracted data, and wrote and edited the manuscript. All authors contributed to the article and approved the submitted version.

## Funding

We would like to thank Bodywhys, The Eating Disorder Association of Ireland, for their generous support in financing the publication of this research.

## Conflict of Interest

The authors declare that the research was conducted in the absence of any commercial or financial relationships that could be construed as a potential conflict of interest.

## Publisher's Note

All claims expressed in this article are solely those of the authors and do not necessarily represent those of their affiliated organizations, or those of the publisher, the editors and the reviewers. Any product that may be evaluated in this article, or claim that may be made by its manufacturer, is not guaranteed or endorsed by the publisher.
