# Supplementary material for: Systematic Review of Digital Interventions for Adolescent and Young Adult Women's Body Image
Source: Front Glob Womens Health. 2022 Mar 17;3:832805. doi: 10.3389/fgwh.2022.832805 (PMC8982933; doi:10.3389/fgwh.2022.832805)
Supplement: Supplementary file 1 [file Data_Sheet_1.docx]

Supplementary Material

# 1 Table 1.

*Search terms used for systematic review*

| Population | | Intervention type | | Outcome: Body Image |
| --- | --- | --- | --- | --- |
| Adolescent  OR  Youth  OR  Young person  OR  Emerging adult*  OR  Young adult  OR  student | Women  OR  girl  OR  female | Digital  OR  Internet  OR  Ehealth  OR  e-health  OR  electronic  OR  Mobile  OR  smartphone  OR  computer-based  OR  online  OR  web-based  OR  playable device  OR  social media  OR  gamif*  OR  application  OR  mobile app  OR  computer application  OR  mobile learning  OR  mobile health  OR  tablet computer  OR  Elearning  OR  digital game-based learning  OR  mobile phone  or  internet-based | intervention  OR  program*  OR  training  OR  support | Body dissatisfaction  OR  Body appreciation  OR  Body satisfaction  OR  Body image  OR  Body esteem  OR  Body positivity  OR  Positive body image  OR  Body acceptance  OR  Body evaluation  OR  Body checking  OR  Body shame  OR  Body surveillance  OR  Body image concern  OR  Body image disturbance  OR  Body image distress  OR  Body image preoccupation  OR  Body image attitudes  OR  Body image anxiety  OR  Body awareness  OR  Weight dissatisfaction  OR  Weight concerns  OR  Shape concerns |
|  |  |  |  |  |

*Note: Columns represent “AND” searches where at least one term from each column was required. “OR” indicates that any of these key words are eligible for inclusion.*

**1.2 Sample Search conducted on Web of Science**

Web of Science UCD (N=409)

((((TS=(adolescent OR "young adult" OR "emerging adult" OR youth OR "young person" OR student)) AND TS=(woman OR female OR girl)) AND TS=("body dissatisfaction" OR "body satisfaction" OR "body appreciation" OR "body acceptance" OR "body image flexibility" OR "body positive" OR "body positivity" OR "positive body image" OR "negative body image" OR "body image disturbance" OR "body image distress" OR "body image concerns" OR "body image preoccupation" OR "body image awareness" OR "body esteem" OR "weight esteem" OR "weight dissatisfaction" OR "weight concerns" OR "shape concerns" OR "weight preoccupation" OR "body evaluation" OR "body surveillance" OR "body anxiety" OR "body checking" OR "body shame")) AND TS=(digital OR online OR "app-based" OR "mobile app" OR "smartphone app" OR "computer application" OR "mobile application" OR "technological-device" OR "computer-based" OR "internet-based" OR "web-based" OR "technolog*" OR "playable device" OR "smartphone" OR "social media" OR internet OR elearning OR "e-learning" OR "digital game-based learning" OR "mobile learning" OR "mobile health" OR "tablet computers" OR electronic)) AND TS=(intervention OR program* OR prevention OR preventive OR training OR support)

**2 Table 2.**

*Full Cochrane Quality Assessment of Studies Included in the Review*

| **Study** | **Selection bias - Random sequence generation** | **Selection bias - Allocation concealment** | **Performance bias - Blinding of participants** | **Detection bias - Blinding of outcome assessment** | **Attrition bias - Incomplete outcome data** | **Reporting bias** |
| --- | --- | --- | --- | --- | --- | --- |
| Atkinson & Diedrichs (2021) | **Medium risk:** Lab sessions were randomized: quasi-experimental design used. | **High Risk:** Doesn't mention using a random numbers table or concealing the allocation sequence from those enrolling participants in the study | **Low risk:** "Participants were blind to conditions" | **High risk:** "Researchers were not blinded to conditions" | **Low risk:** Low attrition rates: at baseline n=202, post-test = 192, follow up = 190. | **Low risk:** All outcomes appear to be reported on |
| Winzelberg et al. (2000) | **High risk:** No mention of randomized allocation | **High Risk:** Doesn't mention using a random numbers table or concealing the allocation sequence from those enrolling participants in the study | **High risk:** No blinding procedures described. Study was advertised with an "entrance requirement was a desire to improve body image satisfaction" | **High risk:** No description of how information was concealed from researchers | **Low risk:** 8 dropped out during the pre—post (13%), 8 more participants did not complete the 3-month follow-up (26%). | **Low risk:** All outcomes appear to be reported on. |
| Bruning Brown et al. (2004) | **High risk:** No mention of randomized allocation | **High Risk:** Doesn't mention using a random numbers table or concealing the allocation sequence from those enrolling participants in the study | **Medium risk:** No blinding procedures described. Some measures taken to conceal study from participants "To reduce potential cross- contamination, students in the intervention were instructed not to discuss it with other students". | **High risk:** No description of how information was concealed from researchers | **High risk:** No information provided on dropouts or attrition. | **High risk:** No effect sizes reported, although these are discussed in the discussion. No information on attrition or whether ITT (intention to treat) or PP (per protocol/completer analyses) was used. In the discussion, authors state "Although other measures did not reach statistical significance, the intervention effect sizes were promising", however, effect sizes were not reported. |
| Halliwell et al. (2011) | **Low risk:** Randomly allocated, through a computer-generated randomization table (www.randomization.com) | **Low risk:** Randomly allocated, through a computer-generated randomization table (www.randomization.com) | **Medium risk:** No blinding procedures described. Some measures taken to conceal study from participants. Study was described to the participants as ‘an evaluation of attitudes towards health, appearance and magazines’. The girls were asked not to talk to each other during the first part of the lesson. | **High risk:** No description of how information was concealed from researchers | **Low risk:** There does not appear to have been any drop out- at least these are not reported on. | **Low-medium risk:** All outcomes appear to be reported on. However, no pre-post assessment of body image before and after viewing the videos and experimental condition. Only compared post-viewing scores and used a trait level of body dissatisfaction to indicate baseline differences in body dissatisfaction across groups. |
| Serdar et al (2014) | **Low risk:** "Participants were randomized and notified via email of their inclusion status and group assignment." | **High Risk:** Doesn't mention using a random numbers table or concealing the allocation sequence from those enrolling participants in the study | **High risk:** No blinding procedures described, participants were aware of their group assignment. "Participants were randomized and notified via email of their inclusion status and group assignment." | **High risk:** No description of how information was concealed from researchers | **Low-medium risk**: "Among program participants, 26.6% did not attend any sessions after randomization". | **High risk:** Authors note that "Nonsignificant results are not reported here" and only significant results presented in tables. |
| Kosinski (2019) | **Low risk:** "Participants were randomly assigned to either the neutral condition or the EC condition". | **Low risk:** Group assignments were predetermined by subject number, odd numbered participants were assigned to the EC condition, even numbered participants were assigned to the neutral condition | **Medium risk:** "The experiment was presented as dealing with the relationship between emotional state and performance in a self- related visual memory game (no reference to body dissatisfaction or eating behavior was included in the presentation of the study)". "Participants completed the experiment individually" and so it is unlikely they were aware of other conditions. | **High risk:** No description of how information was concealed from researchers | **Low risk:** "No participants were excluded or dropped out, and there were no missing data." | **Low risk:** All outcomes appear to be reported on |
| Low et al.(2006) | **Low risk:** "After baseline assessment, women were randomized into four groups" | **High Risk:** Doesn't mention using a random numbers table or concealing the allocation sequence from those enrolling participants in the study | **High risk:** No blinding procedures described. Participants were "Invited to participate in an online pro- gram designed to “deliver information about nutrition, exercise, and diet, and improve body image.” and so were aware of purposes of the study. | **High risk:** No description of how information was concealed from researchers | **Low risk:** "Of the 72 original participants, there were seven non-compliers (9.7%) who never logged on to the program, and who were excluded from analyses. An additional four participants could not be located at long-term follow-up, resulting in a 6% attrition rate." | **High risk:** Authors report completer analyses as main output and only briefly referring to ITT analyses. No effect sizes were reported. Authors state in the discussion: "Student Bodies appears to have reduced risk for eating and body image concerns in this sample of college women, and these effects continued over long-term follow-up". However, interaction effects were not observed, and there was no discussion of unexpected main effects of time or that most of the effects they reported on were not significant (p=.06+). |
| Franko et al. (2012) | **Low risk:** Random assignment used | **Medium-Low risk:** "Program order was counterbalanced". | **High risk:** It is likely that participants could figure out which condition they were assigned to "We told participants that they could be randomly assigned to the control condition; it is not likely that the control websites were interpreted as treatment, because they focused on science" | **High risk:** No description of how information was concealed from researchers | **Low risk:** "Fifty-nine participants (92%) completed both pre- and posttests; 51 (80%) completed all three assessments (pre-, post-, and 3-month follow-up)." ITT analyses used. | **Low risk:** All outcomes appear to be reported on |
| Mulgrew et al (2019) | **Low risk:** Random allocation used | **High Risk:** Doesn't mention using a random numbers table or concealing the allocation sequence from those enrolling participants in the study. | **High risk:** "Participants were randomized and notified via email of their inclusion status and group assignment". | **High risk:** No description of how information was concealed from researchers | **Low-medium risk:** Of the 117 who commenced, 81 completed post intervention assessments (30% attrition) | **Medium risk:** Only reported PP analyses. But most analyses are presented. |
| Toole & Craighead (2016) | **Low risk:** "Participants were randomly assigned to either the intervention or waitlist control group at the first visit" | **High Risk:** Doesn't mention using a random numbers table or concealing the allocation sequence from those enrolling participants in the study | **Medium risk:** True aims of the study were concealed from participants, although blinding procedures to condition assignments were not described. "The study was described as an evaluation of the effects of meditation on aspects of psychological well-being. Participants were not told at the outset that the study targeted self-compassion or body image concerns and there was no screening for body image concerns or eating symptoms." | **High risk:** No description of how information was concealed from researchers | **Low risk:** 4.6% Attrition. ITT analyses conducted | **Low risk:** All outcomes appear to be reported on |
| Franko et al (2013) | **Low risk:** "Classes were randomly assigned to either the intervention or the control condition, with 2–3 classes per school in each condition" | **High Risk:** Doesn't mention using a random numbers table or concealing the allocation sequence from those enrolling participants in the study. | **High risk:** No blinding procedures described although given this was a school study it is unlikely that students were blinded to other conditions. No specific mention of what the study advertised in terms of information sheet. | **High risk:** No description of how information was concealed from researchers | **Medium-low:** Completers: 86/113 Attrition =24%. "Intervention effects were explored on an intent-to-treat basis," | **Low risk:** All outcomes appear to be reported on |
| Matheson et al. (2020) | **Low risk:** "Children were randomly assigned to one of three viewing conditions using the randomization by minimization function in Qualtrics". | **Low risk:** Children were randomly assigned to one of three viewing conditions using the randomization by minimization function in Qualtrics. | **High risk:** No blinding procedures described although given this was a school study it is unlikely that students were blinded to other conditions. No specific mention of what the study advertised in terms of information sheet. | **High risk:** No description of how information was concealed from researchers | **Low risk:** Attrition was minimal (8/1329 =.006%) but unclear if this was for both genders and age groups | **Low risk:** All outcomes appear to be reported on |
| Alleva et al. (2018) | **Low Risk:** Participants were randomized into one of 2 groups | **Low risk:** "indicated their randomly assigned condition, determined by a randomization list (Graph Pad Software, 2012) with a 1:1 allocation ratio" | **Medium risk:** No specific blinding procedures described, however participants were not given any information about the content of the programs. Participants were told that the researchers were creating a well-being magazine for women in England and wanted their opinion about the kinds of advertisements they like. | **High risk:** No description of how information was concealed from researchers | **Low - medium risk:** 28% at 1 week 29.9% at 1-month - possibly due to technical error | **Low risk:** All outcomes appear to be reported on |
| Fuller-Tyszkiewicza et al. (2019) | **Low Risk.** Participants were randomized into one of 2 groups "The online survey was programmed to randomize participants to the intervention or wait-list control group after completing this baseline survey." | **Low risk:** "Online survey was programmed to randomize participants to the intervention or waitlist control group" | **High risk:** No blinding procedures described. "The study was advertised via noticeboards and participant pools for students at three universities in Australia, as well as via advertising on websites of eating disorder organizations and services." | **High risk:** No description of how information was concealed from researchers | **High risk:** 63% attrition at post-test for intervention; 29% WL control | **Low risk:** All outcomes appear to be reported on |
| Matheson et al. (2021) | **Low Risk** Participants were randomized into one of three conditions: body image playable, body image social networking posts (i.e. static images of the body image playable messages; control 1) or an ocean conservation playable (control 2). | **Low risk:** They were randomized to conditions by the research agency using a computer algorithm. | **Low risk:** Participants were blinded to study objectives, hypotheses and their condition. | **High risk:** No description of how information was concealed from researchers | **Medium/Low**: ITT 31% attrition rate overall - higher for girls but not specified | **Low risk:** ITT analyses conducted. "The total proportion of missing data were 15% and 15 imputed data sets were created using type 1 partial mean matching for continuous measures, and logistic regression for the call to action." All outcomes appear to be reported |

**3 Figure 1. Reviewer screening summary depicting level of agreement in decisions regarding inclusion/exclusion.**


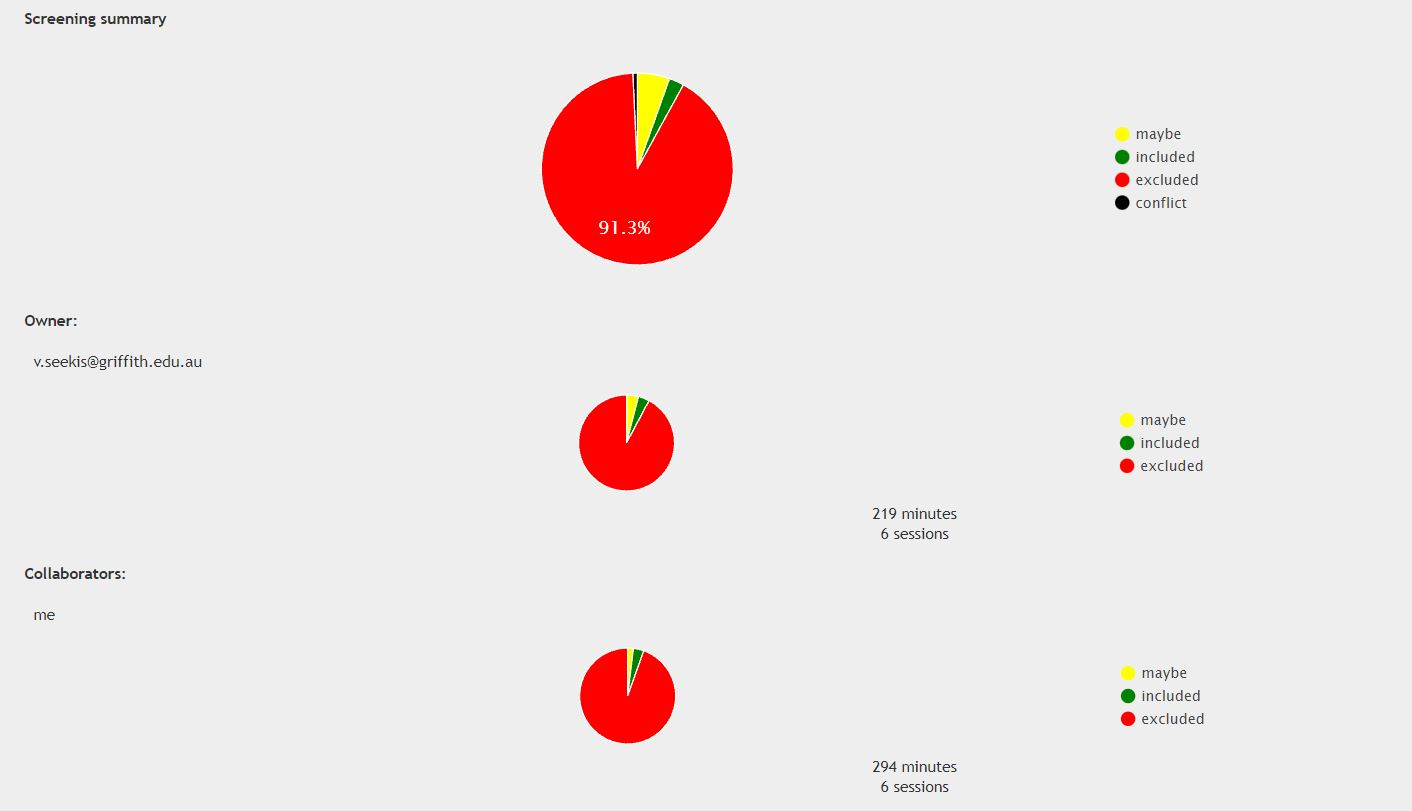
**
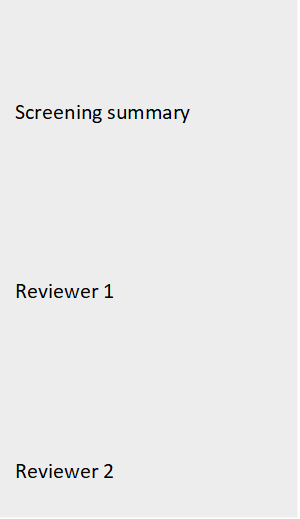
**

Note: Screenshot of screening summary in Rayaan software, which depicts the level of overlap between reviewer 1 and 2 for studies screened for inclusion/exclusion. There was a 91.3% overlap in studies screened for inclusion/exclusion by reviewers.

**4 Modifications to the Review Protocol Preregistered on the Prospero Website**

The study protocol was preregistered on 5^th^ November on the Prospero website <https://www.crd.york.ac.uk/prospero/display_record.php?RecordID=281435>

Two minor modifications were made to this review protocol which, for transparency are listed below

1. Rayyan review software was used instead of Covidence software
2. Given the exploratory nature of the field, we stated in the protocol, that we would potentially include secondary prevention programs (i.e. those that target ‘at risk groups for poor body image’) as a subset/subgroup analysis. This condition was stipulated prior to conducting the review in case insufficient numbers of universal interventions were observed and the search had to be expanded; however, given that there was a considerable number of universal interventions that met inclusion criteria, we opted to focus on universal prevention programs only as per our main inclusion/exclusion criteria. A list of studies that were excluded because they were secondary prevention interventions that targeted ‘at risk’ groups are listed below.

**5 List of Shortlisted Studies that were Excluded and Rationale for Exclusion.**

**Excluded because participants were ‘At risk’ groups [i.e., were identified as having high body dissatisfaction or scored above threshold on body dissatisfaction scale]**

- A preliminary trial of an online dissonance-based eating disorder intervention. (Green et al, 2018)
- Internet‐delivered eating disorder prevention: A randomized controlled trial of dissonance‐based and cognitive‐behavioral interventions (Chithambo et al. 2017)
- Online imagery rescripting among young women at risk of developing an eating disorder: A randomized controlled trial (Zhou & Pennesi, 2020)
- Reducing risk factors for eating disorders: Comparison of an Internet- and a classroom-delivered psychoeducational program (Zabinski et al 2001)
- Cognitive Behavioral Training Using a Mobile Application Reduces Body Image-Related Symptoms in High-Risk Female University Students: A Randomized Controlled Study (Cerea et al.,2021)
- Prevention of Eating Disorders in At-risk College-Age Women (Taylor et al 2006)
- Maintenance of Internet-based prevention: A randomized controlled trial (Jacobi et al. 2007)
- Effects of an Internet-based intervention for subthreshold eating disorders: A randomized controlled trial (Jacobi et al. 2012)
- Student bodies: psycho-education communities on the web. (Dev et al 1999)
- Internet-delivered targeted group intervention for body dissatisfaction and disordered eating in adolescent girls: A randomized controlled trial (Heinicke et al. 2007)
- Effectiveness of an Internet Dissonance-Based Eating Disorder Prevention Intervention Among Body-Dissatisfied Young Chinese Women (Luo et al., 2021)
- Clinician-led, peer-led, and internet-delivered dissonance-based eating disorder prevention programs: Effectiveness of these delivery modalities through 4-year follow-up. (Stice et al. 2017)
- A preliminary trial of a prototype internet dissonance-based eating disorder prevention program for young women with body image concerns (Stice et al., 2012)

**Excluded because participants reported an eating disorder diagnosis**

- Media Smart-Targeted: Diagnostic outcomes from a two-country pragmatic online eating disorder risk reduction trial for young adults (Wilksch et al. 2018)
- Online prevention of disordered eating in at-risk young-adult women: A two-country pragmatic randomized controlled trial SimonOnline prevention of disordered eating in at-risk young-adult women: A two-country pragmatic randomized controlled trial (Wilksch et al. 2018)
- Imagery rescripting and cognitive dissonance: A randomized controlled trial of two brief online interventions for women at risk of developing an eating disorder (Pennesi & Wade, 2018)
- Comparison of internet and face-to-face delivery of a group body image and disordered eating intervention for women: A pilot study (Gollings & Paxton, 2006)

**Excluded because intervention was not (fully) digital**

- A controlled intervention to promote a healthy body image, reduce eating disorder risk and prevent excessive exercise among trainee health education and physical education teachers (Yager & O’Dea, 2010)
- Understanding and promoting treatment-seeking for eating disorders and body image concerns on college campuses through online screening, prevention and intervention
- Comparison of a gratitude-based and cognitive restructuring intervention for body dissatisfaction and dysfunctional eating behavior in college women. (Wolfe et al., 2017)
- Reducing risk factors for eating disorders: Comparison of an Internet- and a classroom-delivered psychoeducational program (Celio et al., 2000)

**Excluded because no gender breakdown of body image outcomes were provided**

- BodiMojo: Efficacy of a Mobile-Based Intervention in Improving Body Image and Self-Compassion among Adolescents (Rodgers et al., 2018)
- Teaching adolescents about changing bodies: Randomized controlled trial of an Internet puberty education and body dissatisfaction prevention program (Cousineau et al., 2010)

**Excluded because studies did not contain a control group/were a study protocol**

- Adaption and evaluation of an Internet-based prevention program for eating disorders in a sample of women with subclinical eating disorder symptoms: A pilot study (Volker et al., 2014)
- Efficacy and cost-effectiveness of Internet-based selective eating disorder prevention: study protocol for a randomized controlled trial within the ProHEAD Consortium. (Bauer et al., 2019)
